# Granuloma Following Tracheostomy Closure: An Overlooked Cause of Chronic Cough

**DOI:** 10.7759/cureus.79127

**Published:** 2025-02-16

**Authors:** Joana Dias, André Sousa-Machado, Sara Costa, Luís Meireles, Sandra Castro

**Affiliations:** 1 Otolaryngology - Head and Neck Surgery, Unidade Local de Saúde de Santo António, Porto, PRT

**Keywords:** bronchoscopy, decannulation, granuloma, persistent cough, tracheostomy

## Abstract

Tracheostomy is a life-saving procedure often performed in cases of airway obstruction, prolonged mechanical ventilation, or neurological conditions affecting respiratory function. Although decannulation is generally safe, delayed complications such as tracheal granulomas can occur and may present with nonspecific symptoms, leading to delays in diagnosis.

This report describes the case of a 45-year-old previously healthy female who developed a persistent dry cough and positional shortness of breath six months after tracheostomy closure. The patient was initially admitted with bilateral vocal cord paralysis following SARS-CoV-2 infection, resulting in tracheostomy. After support treatment with corticosteroid therapy, decannulation was performed 28 days later. During follow-up, the patient developed a refractory dry cough that was worse in the supine position.

CT scan revealed a round, polypoid lesion extending into the tracheal lumen above the carina. The lesion was identified as a tracheal granuloma and was successfully excised via bronchoscopy, resulting in complete symptom resolution.

This case highlights the need to consider tracheal granuloma in patients presenting with persistent respiratory symptoms after tracheostomy decannulation. Early recognition, appropriate imaging, and timely intervention are crucial for symptom relief and prevention of further complications. Bronchoscopic excision remains a safe and effective treatment option.

## Introduction

Tracheostomy has been a cornerstone of airway management, with its indications evolving alongside advancements in critical care medicine [1]. While early complications such as bleeding and infection are well-documented, delayed complications, including tracheal granulomas, may remain underrecognized despite their significant limitation on a patient's daily life [2]. The presence of granulation tissue, a precursor to granuloma formation, has been linked to mechanical irritation from the tracheostomy tube or prolonged stoma healing [3]. Early identification and intervention for post-decannulation complications, such as granulomas or stenoses, are critical to prevent more severe airway compromise and ensure optimal outcomes [4].

## Case presentation

A 45-year-old previously healthy female presented to the emergency department with a four-week history of dry cough, progressive dyspnea on mild exertion, and dysphonia that had developed over the course of one day. The patient tested positive for SARS-CoV-2 and subsequently experienced worsening respiratory symptoms. An otolaryngology consultation revealed bilateral vocal cord paralysis, requiring urgent surgical tracheostomy using a Bjork flap to secure the airway and relieve respiratory distress.

During her hospitalization, the patient received corticosteroid therapy, which resulted in progressive clinical improvement.

After 28 days, flexible endoscopy confirmed appropriate vocal cord mobility, allowing the decannulation process to begin. An uncuffed tube was placed in the stoma, and the tracheostomy appliance was capped. The patient remained comfortable with the capped tube for 24 hours, after which the tube was removed. The site was then covered with gauze, allowing the tracheostomy to heal by secondary intention. One month after decannulation, the tracheostomy site had completely closed. During this time, other than mild paresthesias and discomfort at the cervical scar, the patient had no other complaints, including cough.

**Figure 1:**
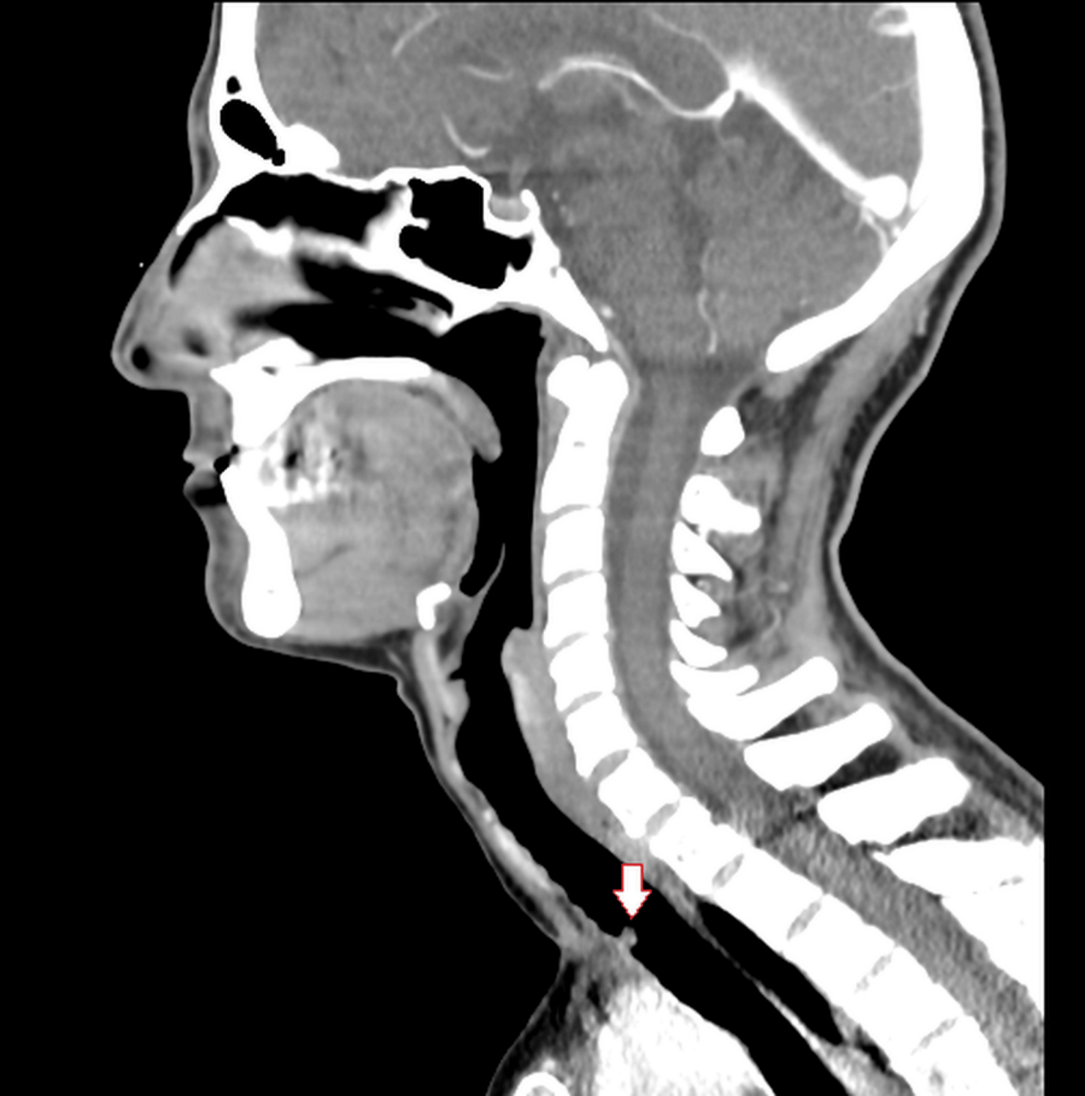
Sagittal computed tomography (CT) image showing an exophytic lesion within the tracheal lumen (arrow) located 7 cm above the tracheal carina

At the 3three-month otolaryngology follow-up, the patient reported a persistent, irritating dry cough that was unresponsive to medical treatment. The cough progressively worsened, particularly in the supine position, and began to interfere with her daily activities and sleep quality. At that time, a thoracic and pharyngolaryngeal computed tomography (CT) scan was performed due to the persistent symptoms. The imaging revealed a round, polypoid lesion protruding into the tracheal lumen above the carina, causing partial airway obstruction (Figure 1).

**Figure 2:**
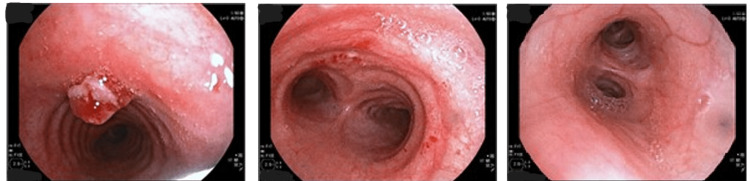
Polypoid and exophytic lesion protruding into the tracheal lumen 7 cm above the tracheal carina

Bronchoscopic examination under local anesthesia with topical lidocaine and sedation with midazolam and fentanyl confirmed the lesion as a tracheal granuloma 7 cm above the tracheal carina (Figure 2). The granuloma was excised bronchoscopically, and histopathological analysis confirmed its benign nature as granulation tissue.

Following excision, the patient reported immediate symptom improvement, including resolution of cough and dyspnea. Follow-up over the next year confirmed the patient remained asymptomatic, with no recurrence of granuloma or airway complications.

## Discussion

Granulomas are among the most common late complications following tracheostomy, resulting from excessive granulation tissue formation at the site of the tracheostomy [5]. These lesions are thought to arise due to chronic mechanical irritation or prolonged inflammation at the stoma site, which stimulates abnormal healing processes [6]. In this case, the granuloma was located 7 cm above the tracheal carina, at the distal end of the tracheostomy tube. In such cases, cuff-related trauma, foreign body reaction to the tracheostomy tube material, and accumulation of secretions due to inadequate suctioning may contribute to granuloma formation.

Clinically, granulomas can manifest with nonspecific respiratory symptoms such as persistent dry cough, dyspnea, or voice changes, as in the presented case [7]. These symptoms often overlap with other respiratory conditions, potentially delaying diagnosis. Imaging modalities, particularly CT scans, play a crucial role in visualizing exophytic lesions in the tracheal lumen, guiding further diagnostic and therapeutic approaches [8].

Bronchoscopy remains the gold standard for both the diagnosis and management of tracheal granulomas. It provides direct visualization of the lesion and allows for minimally invasive excision, which often results in immediate symptomatic relief and an excellent prognosis [9]. Delayed recognition of these lesions can lead to significant morbidity, including airway obstruction or secondary infections. Complications such as distal tracheal granulation tissue have also been reported, emphasizing the need for individualized surgical and therapeutic approaches [10,11].

Peristomal complications, particularly in pediatric cases, highlight the diversity of presentations and the importance of timely intervention to prevent long-term sequelae [9]. This case emphasizes the importance of considering tracheal granulomas in patients with persistent respiratory symptoms, particularly following tracheostomy or prolonged intubation, as timely intervention is critical to restoring airway patency and improving quality of life [4].

This case highlights the importance of recognizing tracheal granuloma as a differential diagnosis in patients presenting with persistent respiratory symptoms following tracheostomy decannulation. Early diagnosis through imaging and bronchoscopy is vital for effective management. Bronchoscopic excision is a safe and effective treatment modality, often leading to rapid clinical improvement. Raising awareness about this rare yet impactful complication is essential to improving outcomes in patients undergoing tracheostomy and decannulation.

## Conclusions

Imaging plays a critical role in evaluating chronic, positional cough, particularly when symptoms are refractory to conventional treatment. Objective and direct tools such as flexible laryngoscopy are essential in assessing post-tracheostomy patients. They provide a dynamic, real-time evaluation of upper airway function, aiding in the detection of subtle structural abnormalities, vocal cord dysfunction, or persistent inflammation that may contribute to respiratory symptoms. When a structural airway abnormality is suspected, a combination of nasofibrolaryngoscopy, CT imaging, and bronchoscopy is essential for accurate diagnosis and appropriate management. Bronchoscopic excision remains a safe and effective diagnostic and therapeutic approach, often leading to rapid symptom resolution and excellent long-term outcomes.

Raising awareness of this uncommon complication is crucial for improving clinical decision-making in post-tracheostomy follow-up. A structured approach incorporating objective airway assessments - flexible laryngoscopy, imaging, and bronchoscopy - is key to timely recognition and intervention, ultimately enhancing patient outcomes.
